# Phenotype Correlations With Pathogenic DNA Variants in the *MUTYH* Gene: A Review of Over 2000 Cases

**DOI:** 10.1155/2024/8520275

**Published:** 2024-09-27

**Authors:** Monica Thet, John-Paul Plazzer, Gabriel Capella, Andrew Latchford, Emily A. W. Nadeau, Marc S. Greenblatt, Finlay Macrae

**Affiliations:** ^1^ Melbourne Medical School The University of Melbourne, Parkville, Victoria, Australia; ^2^ Department of Medicine University of Melbourne Royal Melbourne Hospital, Parkville, Australia; ^3^ Department of Colorectal Medicine and Genetics Royal Melbourne Hospital, Parkville, Australia; ^4^ Hereditary Cancer Program Catalan Institute of Oncology IDIBELL Hospitalet de Llobregat, Barcelona, Spain; ^5^ Centro de Investigación Biomédica en Red de Cáncer (CIBERONC), Madrid, Spain; ^6^ Polyposis Registry St Mark's Hospital, Harrow, UK; ^7^ Department of Surgery and Cancer Imperial College, London, UK; ^8^ Department of Medicine University of Vermont Larner College of Medicine, Burlington, Vermont, USA

## Abstract

*MUTYH*-associated polyposis (MAP) is an autosomal recessive disorder where the inheritance of constitutional biallelic pathogenic *MUTYH* variants predisposes a person to the development of adenomas and colorectal cancer (CRC). It is also associated with extracolonic and extraintestinal manifestations that may overlap with the phenotype of familial adenomatous polyposis (FAP). Currently, there are discrepancies in the literature regarding whether certain phenotypes are truly associated with MAP. This narrative review is aimed at exploring the phenotypic spectrum of MAP to better characterize the MAP phenotype. Literature search was conducted to identify articles reporting on MAP-specific phenotypes. Clinical data from 2109 MAP patients identified from the literature showed that 1123 patients (53.2%) had CRC. Some patients with CRC had no associated adenomas, suggesting that adenomas are not an obligatory component of MAP. Carriers of the two missense founder variants, and possibly truncating variants, had an increased cancer risk when compared to those who carry other pathogenic variants. It has been suggested that somatic G:C > T:A transversions are a mutational signature of MAP and could be used as a biomarker in screening and identifying patients with atypical MAP, or in associating certain phenotypes with MAP. The extracolonic and extraintestinal manifestations that have been associated with MAP include duodenal adenomas, duodenal cancer, fundic gland polyps, gastric cancer, ovarian cancer, bladder cancer, and skin cancer. The association of breast cancer and endometrial cancer with MAP remains disputed. Desmoid tumors and congenital hypertrophy of the retinal pigment epithelium (CHRPEs) are rarely reported in MAP but have long been seen in FAP patients and thus could act as a distinguishing feature between the two. This collection of MAP phenotypes will assist in the assessment of pathogenic *MUTYH* variants using the American College of Medical Genetics and the Association for Molecular Pathology (ACMG/AMP) Variant Interpretation Guidelines and ultimately improve patient care.

## 1. Introduction

Gastrointestinal (GI) polyposis syndromes are characterized by tens to thousands of polyps, of which adenomatous polyposis is the most common type. Adenomatous polyps are precancerous lesions which, if undetected, can develop into colorectal cancer (CRCs). To date, numerous types of inherited adenomatous polyps have been discovered. While each inherited form harbours adenomatous polyps, the quantity of polyps and the extracolonic manifestations can vary depending on the type.

*MUTYH*-associated polyposis (MAP) is one of these adenomatous polyposis syndromes. First described in 2002 by Al-Tassan et al., it is an autosomal recessive disorder where one inherits constitutional biallelic pathogenic variants in the *MUTYH* gene [1]. These pathogenic variants predispose individuals to the development of adenomatous polyps and CRCs [1–5]. *MUTYH* is one of the base excision repair (BER) genes located on chromosome 1 (1p34.3–p.32.1) and is involved in the repair of oxidative damage. The two founder variants, p.(Tyr179Cys) and p.(Gly396Asp), are by far the most common pathogenic *MUTYH* variants in Caucasian populations [1, 4–7]. Because of alternative start codons and alternative splicing, the amino acid positions of these codons have not been agreed upon in the literature. The most commonly cited transcript corresponds to p.Tyr179Cys and p.Gly396Asp, with allele frequencies in European populations of 0.15% and 0.3%, respectively, according to the ClinVar database at the National Centre for Biotechnology Information (NCBI).

Constitutional pathogenic *MUTYH* variants account for 30%–40% of cases with adenomatous polyposis where a constitutional variant in the adenomatous polyposis coli (*APC*) gene is not detected [4–7]. There are also reported cases where polyps are absent [8–10], and therefore, the term MAP may not represent the entire population of patients with constitutional biallelic pathogenic *MUTYH* variants. “*MUTYH*-associated tumor syndrome,” an alternative to MAP, has been proposed to better represent the broad phenotypic range associated with pathogenic *MUTYH* variants [11].

MAP is also associated with several extracolonic and extraintestinal manifestations, and these may overlap with, but not duplicate, the phenotype of familial adenomatous polyposis (FAP) [12]. A comprehensive characterization of the MAP phenotype will improve the diagnosis and treatment of MAP. Moreover, it will also contribute to the development of *MUTYH*-specific American College of Medical Genetics and the Association for Molecular Pathology (ACMG/AMP) Variant Interpretation Guidelines. The well-established approach by ClinGen Variant Curation Expert Panels to the determination of pathogenicity of variants in genes, including *MUTYH*, requires a clear description of the phenotype of any disease or syndrome attributable to pathogenic variants in the gene under consideration. This then allows for statistical associations within and between families of affected individuals, defined by such descriptions, with gene variants, enabling substantiation of the pathogenicity of the variant in the five-class system derived from a Bayesian likelihood analytic approach. This narrative review importantly supports this clear definition of phenotype by exploring the phenotypic spectrum of MAP described in the literature, including colonic, extracolonic, and extraintestinal manifestations, and is aimed at better describing the syndrome-specific phenotype of *MUTYH*-associated tumor syndrome.

## 2. Methods

**Figure 1:**
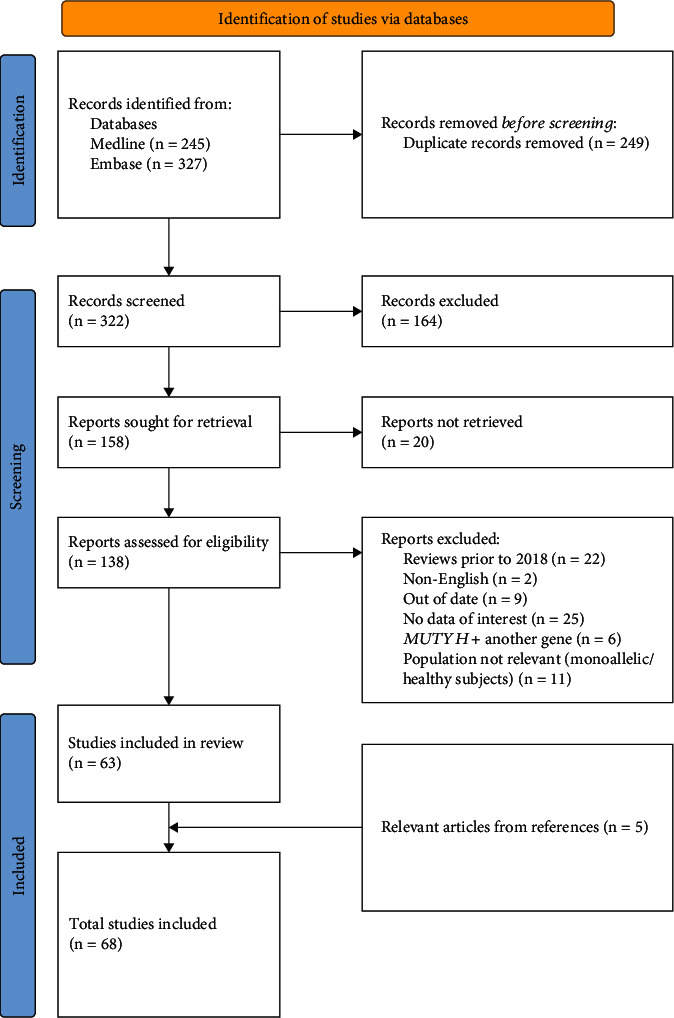
PRISMA flow chart illustrating the systematic approach for study inclusions through the stages of this narrative review.

To find articles describing the phenotypes of biallelic pathogenic *MUTYH* variants, the following key search terms were used: MUTYH, MUTYH-associated polyposis, base excision repair, adenomatous polyp, colorectal cancer, colorectal adenoma, gastrointestinal cancer, extracolonic cancer/tumor, extraintestinal cancer/tumor, phenotypic variability, and genotype-phenotype correlation (see Supporting Information: Table S1 for search strategy and results). All relevant references within these articles were also included. The following exclusion criteria were applied: review articles prior to 2018 (except those that are highly cited in the literature), non-English articles, articles that are out-of-date or without data of interest, irrelevant populations (monoallelic or healthy controls), and articles looking at interactions between *MUTYH* and other genes (Figure 1). The cut-off year of 2018 was chosen because comprehensive reviews for *MUTYH* published around and after 2018 also collected relevant information from studies conducted prior to 2018.

**Figure 2:**
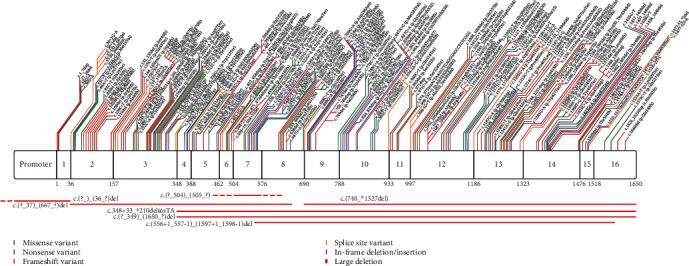
Location and type of pathogenic variants in *MUTYH* identified in the literature, ClinVar, and LOVD.

Pathogenic *MUTYH* variants from the literature, ClinVar and the Leiden Open Variation Database (LOVD) were mapped to the gene in Figure 2. A five-tier system is currently used to classify variants into one of the following categories: pathogenic, likely pathogenic, variant of uncertain significance (VUS), likely benign, and benign. If a variant had been classified under more than one category, the higher classification was used. For instance, if a variant was classified as both pathogenic and likely pathogenic, it was considered as pathogenic and thus included in Figure 2. The reference transcript NM_001128425.2 was used to describe pathogenic variants.

## 3. Results and Discussion

A total of 234 pathogenic variants were identified in the literature, ClinVar, and LOVD. Of these, 77 were reported in articles describing the phenotypic characteristics of the affected individuals. The remaining 156 variants were not identified in the literature but were reported in ClinVar, LOVD, or in both ClinVar and LOVD. Frameshift (*n* = 87) and nonsense (*n* = 71) variants were the most common types of pathogenic *MUTYH* variants (Table 1). Pathogenic missense variants can occur throughout the entire length of the protein and are not confined to one region or domain.

Clinical features of MAP patients reported in the literature are summarized in Table S2. Population inclusion criteria varied across the studies. However, patients with adenomas or CRCs who had a pathogenic variant in *APC* and/or mismatch repair (MMR) genes were consistently excluded. Variant analysis was conducted in one of two ways. The first method involved sequencing the entire coding region, with or without exon–intron junctions, to identify variants. The second method involved first sequencing the two pathogenic founder variants, p.(Tyr179Cys) and p.(Gly396Asp). Patients who were heterozygous for one of these two variants then had the entire *MUTYH* gene sequenced to search for a second pathogenic allele. It should be noted that this latter method has a possibility of missing MAP patients with nonfounder pathogenic variants.

The identification of extracolonic and/or extraintestinal manifestations was dependent on the degree of intensity that they were looked for, and as such, not all studies reviewed here included findings about these manifestations. This applies particularly to the index cases in families who are generally symptomatic when compared to other affected family members and hence were subject to more extensive investigation. Overall, the percentage of MAP patients with upper GI findings was calculated based only on the population of patients in the study who underwent gastroscopy. Of the 2109 MAP patients recorded in Table S1, 1123 patients had CRC (53.2%). Nine hundred thirty-six patients underwent upper GI gastroscopy, and 160 of these patients were reported as having duodenal adenomas (17.1%). Three patients had duodenal cancer (0.3%), all of whom had concurrent duodenal adenomas at the time of diagnosis. Summary statistics were not reported for other phenotypes due to incomplete investigation across the studies.

### 3.1. Variant Pathogenicity

In Western populations, up to 70% of MAP patients carry the two pathogenic founder variants, p.(Tyr179Cys) and p.(Gly396Asp), in either homozygosity or compound heterozygosity. Moreover, as many as 93% of MAP patients carry at least one of these two variants [6]. Other pathogenic *MUTYH* variants that may be specific to certain ethnic groups include p.(Tyr104Ter), p.(Glu466Ter), p.(Glu480del), and c.1227_1228dup p.(Glu410GLyfsX43) in Pakistani, Indian, Italian, and Portuguese populations, respectively [4, 13–15]. The variant c.1227_1228dup has also been found in high frequency in North African patients [16–18].

Due to the widely available data for these two founder variants in Western populations, multiple studies have evaluated their pathogenicity. Studies have found that p.(Tyr179Cys) homozygous carriers had earlier disease onset, more severe phenotype, and increased cancer risk when compared to homozygous carriers of p.(Gly396Asp) and compound heterozygous carriers of these two founder variants [9, 10, 19–21]. The risk of CRC was doubled in patients carrying these two founder variants, either in homozygosity or compound heterozygosity, when compared to biallelic carriers of other pathogenic variants [10].

Evidence suggests that the molecular consequences of variants also have varying pathogenicity. Patients with truncating pathogenic variants and in-frame deletions that result in exon skipping have been reported to have a more severe phenotype than patients with missense pathogenic variants [19, 22, 23]. In a study by Nielsen et al. [19] that contained 257 MAP patients, more patients with biallelic truncating pathogenic variants (5 of 11, 45%) appeared to have polyp counts of > 100 in comparison to those with zero (28 of 109, 26%) or one (4 of 28, 15%) truncating pathogenic variants, even though this finding was not statistically significant [19]. Interestingly, the same study also found that none of the patients with two truncating pathogenic variants had fewer than 10 polyps.

Based on this data, it can be inferred that different pathogenic variants result in varying degrees of pathogenicity. Patients with the same pathogenic variant may have varying numbers of polyps, may or may not develop CRC, and may or may not develop other extracolonic phenotypes [6, 7]. This suggests that phenotypic heterogeneity is not governed only by specific pathogenic *MUTYH* variants. Instead, the penetrance of these variants is likely influenced by epigenetic, other genetic, and environmental factors [24].

### 3.2. Age of Diagnosis

The reported age of diagnosis of patients with MAP varied across the studies included in this review, with the median age of index cases at diagnosis ranging from 45 to 58 (Table S1). The youngest case reported was a 13-year-old Caucasian male, compound heterozygous for p.(Tyr179Cys) and p.(Gly396Asp) [2]. He had more than 100 polyps at the time of diagnosis. No CRC was diagnosed at the time, but he developed gastric cancer at the age of 17. Based on the recruitment criteria of this study [2], he had no pathogenic *APC* variant identified and there was no vertical transmission of polyposis in his family. However, his age at diagnosis is unusual and lies well outside the median age range at diagnosis for biallelic *MUTYH* patients, suggesting that there may be other factors contributing to the case.

### 3.3. Somatic G:C > T:A Transversions

In the presence of reactive oxygen species, guanine is converted to 8-oxoguanine (8-oxoG), a compound that mispairs with adenine. The *MUTYH* gene encodes a 535-amino acid DNA glycosylase that identifies and removes adenine bases mispaired with 8-oxyguanine [1]. However, if these A:8-oxoG mispairs are left incorporated in DNA, they lead to somatic G:C > T:A transversions upon replication. These transversions are typically observed in the *APC* gene in adenomas of people with constitutional, biallelic, pathogenic *MUTYH* variants [6, 13, 25–27], as well as in other genes such as *KRAS* [13, 25–28].

Multiple nucleotide substitutions, predominantly in Codons 12, 13, and 61, are prevalent in multiple tumors and CRC [29]. However, only two of these G:C > T:A transversions are at the first (Base 34, results in Gly12Cys) and second (Base 35, results in Gly12Val) nucleotides of Codon 12. These transversions are oncogenic and can frequently occur in both FAP-associated colorectal tumors and sporadic tumors [26, 30]. Interestingly, *KRAS* mutations in MAP tumors are exclusively G:C > T:A transversions at the first G of Codon 12 [25, 26, 28, 30, 31]. The finding of this specific somatic Gly12Cys variant in the *KRAS* gene in all observed patients with constitutional biallelic pathogenic *MUTYH* variants compared to patients with sporadic (13.6%) or FAP-associated adenomas or CRCs (18.5%) is statistically significant (*p* ≤ 0.001 and *p* ≤ 0.002, respectively) [13, 26]. Another recent study that compared the prevalence of somatic mutations in CRCs of patients with constitutional biallelic pathogenic *MUTYH* variants (*n* = 19) to patients without constitutional or somatic pathogenic *MUTYH* variants (*n* = 5364) found that patients with *MUTYH* variants had a significantly higher proportion of somatic *KRAS* G > T transversions at Codon 12 (Gly12Cys) (84% vs. 2.4%; *p* = 2.0 × 10^−23^) [32]. Therefore, this variant could act as a potential biomarker for screening and identifying patients with atypical MAP, that is, without polyps or with less than 10 polyps [31]. Moreover, screening for this variant in extracolonic tumors of MAP patients could help differentiate between tumors that occur sporadically and those that are caused by pathogenic *MUTYH* variants, which allows for a more accurate representation of the MAP phenotypic spectrum. This biomarker could also be used to identify an additional cohort of *MUTYH* carriers whose families would benefit from genetic counselling.

### 3.4. CRC and Colonic Polyps

MAP patients have an increased risk of developing CRC compared to the general population, with approximately two-thirds of MAP patients developing CRC [3, 6, 33, 34]. MAP patients have variable age of diagnosis, polyp count, and polyp type and may have CRC at different locations. CRC is more commonly confined to the right side of the colon, with at least 60% of MAP patients having CRC proximal to the splenic flexure [3, 19, 28, 35–37]. The mean age of CRC diagnosis in MAP patients is 42.3–50 years, and 95% of patients are diagnosed with CRC after the age of 35 [3, 38–40]. The age at diagnosis of CRC in MAP patients is younger than patients with sporadic CRC (68 years) [28, 38, 41]. The youngest reported CRC patient thus far is a 21-year-old female with 36 polyps and compound heterozygous for p.(Tyr179Cys) and c.1147del p.(Ala385ProfsTer23) [3].

Most colonic polyps in MAP patients are adenomas, and polyp counts are variable. It has been found that MAP patients have a higher polyp count and earlier presentation (median age = 47 years) compared to non-MAP patients (median age = 54 years) [40]. While some studies have suggested that it is rare to only have a few adenomas in patients with biallelic pathogenic *MUTYH* variants [2–4, 6, 19], polyps may not be an obligatory phenotype of MAP [10], as the absence of polyps has been noted in multiple studies [8–10, 34]. However, studies that mentioned the absence of polyps were conducted prior to 2010. Since that time, there have been significant advances in endoscopy (high-definition imaging, narrow band imaging, artificial intelligence, etc.) that have facilitated polyp detection. Therefore, if phenotype was ascertained through colonoscopy, it is possible that polyps may have been overlooked due to older endoscopic technologies and these patients may have had a more typical MAP phenotype.

Patient cohorts that have polyp counts between 10 and 99 have been reported to have more carriers of pathogenic *MUTYH* variants when compared to patient cohorts that have a polyp count between 100 and 1000 [2, 4, 6, 25]. However, other studies have found that pathogenic, biallelic *MUTYH* variants occur at equal frequencies in both patient groups [3, 42]. These differences could be due to different methods of ascertainment, including symptomatic index cases and asymptomatic relatives. Although not included in our review, phenotype is likely to be more variable in cohorts identified by panel testing of phenotypes not associated with polyposis or CRC. In addition to high polyp count not being a necessary component of MAP [10], polyp count may also not be a reliable predictor of CRC development. Several studies have suggested that high polyp burden is not associated with the occurrence of CRC [41, 43]. In fact, CRC has developed in patients with fewer than 10 adenomas [41]. These contrasting reports may indicate that polyp count and the development of CRC are not reliable indicators for genetic testing aimed at identifying *MUTYH* variants.

The most frequent polyp type described in MAP is tubular adenomas. Tubulovillous adenomas and, rarely, hyperplastic polyps (HPs) and sessile serrated lesions (SSLs) have also been reported [20, 44–46]. Boparai et al. found that 47% (8/17) of patients had HP and SSL in addition to adenomas [44]. While adenomas in this study harboured both *APC* and *KRAS* mutations with somatic G:C > T:A transversions, HP and SSL only had *KRAS* mutations [44]. G:C > T:A transversions in HP and SSL were more often observed in MAP patients (51 of 73 patients) than the control group with sporadic adenomas (7 of 41 patients; *p* < 0.0001). Data suggests that these polyp types may also be a part of the MAP phenotype. Alternatively, there could be a subset of serrated polyposis syndrome where *MUTYH* is a contributing factor but not the root cause [47]. For example, an unknown interaction between *MUTYH* and another gene could be the cause.

### 3.5. Extracolonic GI Manifestations

Although data on the presence of somatic G:C > T:A transversions in the *KRAS* gene in duodenal lesions in MAP patients is limited [48], by analogy with the colon, the presence of somatic G:C > T:A transversions in duodenal lesions would strongly support the likelihood that duodenal lesions are caused by a deficiency in *MUTYH* activity [48]. The prevalence of duodenal polyps ranged from 17% (26/150) of the cases in Vogt et al., 18% (6/33) of the cases in Aretz et al., and 34% (31/92) of cases in Walton et al. [5, 12, 49]. This variability in prevalence may be because not all MAP patients in these studies underwent endoscopic examination of the upper GI tract. The age at the time of the endoscopic evaluation also varied. Therefore, these estimates concerning the prevalence of duodenal polyps in MAP remain preliminary. It should also be noted that the histopathological characteristics of duodenal lesions were not always described. However, outside of the hamartomatous polyposis syndromes, duodenal polyps other than adenomas are exceedingly rare. In the three studies described above, all 31 patients with duodenal polyps in Walton et al. [49] and 16 of 26 patients in Vogt et al. [12] had histologically confirmed adenomas, and 1 patient had HPs. The polyp type was unknown in the remaining nine patients. Polyp type was not described in Aretz et al. [5].

When assessed using Spigelman staging, the prevalence and severity of duodenal adenomas is lower in MAP than in FAP [12, 27, 49, 50]. The lifetime risk of developing duodenal cancer in MAP is not well established, but it has been reported to be 4%, which is higher than the general population risk [12]. While Spigelman Stage IV is a strong predictor of duodenal cancer development in FAP, this does not seem to be the case for MAP [48–50]. In contrast to patients with FAP, MAP patients have far fewer duodenal adenomas, and many patients have a solitary adenoma [49, 50]. Furthermore, in MAP, progression through the Spigelman classification occurs predominantly based on progression of histological features and not by progression of adenoma count [49, 50]. It has also been found that, despite having lower Spigelman stage, duodenal adenomas associated with MAP have a higher burden of somatic variants in oncogenic driver genes such as *APC* and *KRAS* when compared to FAP-associated adenomas [27]. In a study that performed whole exome sequencing on 10 duodenal adenomas in 5 MAP patients and 10 duodenal adenomas in 4 FAP patients, the rate of *APC* somatic G > T transversions in MAP adenomas (481/716; 67%) was higher than in FAP adenomas (28/225; 12%) (*p* < 2.2 × 10^−16^). MAP duodenal adenomas also had significantly more *KRAS* mutations than FAP adenomas (8/22 vs. 4/38, respectively; *p* < 0.023) [27]. This higher mutation burden could increase duodenal cancer risk in patients with MAP despite the manifestation of duodenal adenomas (number, size, etc.) being less severe. These observations suggest that it may not be appropriate to use Spigelman staging in MAP-associated duodenal adenomas to predict future disease progression. As such, a low duodenal adenoma count may be falsely reassuring to MAP patients [27].

The risk of developing duodenal adenomas in MAP may be associated with specific pathogenic variants. In a multicentre study involving endoscopic surveillance of the duodenum of 394 MAP patients, patients homozygous for the variant p.(Tyr179Cys) were more likely to have adenomas at initial endoscopy when compared to p.(Gly396Asp) homozygotes or p.(Tyr179Cys)/p.(Gly396Asp) compound heterozygotes [50]. Moreover, adenomas in p.(Tyr179Cys) homozygous carriers were more likely to harbour high-grade dysplasia and/or contain villous components (8 of 67, 12%) when compared to the latter two (0 of 62 p.(Gly396Asp) homozygotes; 2 of 55 p.(Tyr179Cys)/p.(Gly396Asp) compound heterozygotes). This finding further supports the above observation that p.(Tyr179Cys) homozygous carriers have a more severe phenotype than others [19].

Gastric cancer in *MUTYH* is rarely reported, with no estimates of standardised incidence ratio (SIR) available. Furthermore, we were unable to find any studies that have conducted molecular studies on gastric cancer in *MUTYH* patients which, if available, could support an association. Gastric cancer in MAP patients has been reported in some studies and has also been reported in *MUTYH* heterozygotes [2, 12, 36, 51, 52]. Therefore, gastric cancer is possibly not associated with MAP. Further studies estimating SIR and molecular profiling should be helpful to address any association. Fundic gland polyps are also described in MAP literature, although whether they are observed more commonly than the general population is not clear [12, 38, 53]. The occurrence of pyloric gland adenomas in MAP has only been reported once, although it has been reported in about 6% of FAP patients [54].

### 3.6. Extraintestinal Manifestations

Many extraintestinal manifestations have been found in MAP patients. However, the extent to which pathogenic *MUTYH* variants are directly responsible for or are associated with these manifestations is not clear. Although controversial, there is some evidence for the associations outlined below, and thus, they are potentially important components of the MAP phenotypic spectrum. This dispute is due to the lack of statistically significant extraintestinal manifestations in MAP patients when compared to the general population.

In a retrospective study that involved 276 patients, the incidence of three extraintestinal malignancies was almost doubled in MAP patients when compared to the general population [12]. The incidence for ovarian (SIR = 5.7; 95%confidence interval [CI] = 1.2–16.7), bladder (SIR = 7.2; 95%CI = 2.0–18.4), and skin cancers (SIR = 2.8; 95%CI = 1.5–4.8) was increased significantly. It is important to note that the population in this study not only included the index patients and their affected relatives with biallelic pathogenic *MUTYH* variants but also their deceased relatives who had confirmed colorectal adenomatous polyps and/or identified pathogenic, biallelic *MUTYH* variants. This selection criteria suggest that the deceased relatives with polyps, who were not genetically tested, might have been included in the study. Because their phenotype was not confirmed via genotype, their inclusion could have led to ascertainment bias. Nevertheless, some of the findings of this study were supported by Win et al., where the risk of bladder cancer and ovarian cancer was increased by 19-fold and 17-fold, respectively [55]. Ovarian, bladder, and skin cancers are therefore most likely associated with MAP given the statistical significance of the occurrence of these cancers in MAP patients compared to the general population.

The association between breast cancer and *MUTYH* is also contested [3, 12, 55, 56]. In one study, 4 of 22 female Dutch MAP patients developed breast cancer, two under age 50. No study has replicated this observation and thus confirmed a significantly increased risk of breast cancer in biallelic *MUTYH* carriers [3]. Moreover, in another study looking at the prevalence of *MUTYH* mutations in 691 breast cancer patients and 812 healthy controls, it was found that none of the participants carried the two founder mutations [56]. However, this study only screened for the two founder mutations and did not sequence the entire gene. Thus, this study could have failed to identify other pathogenic *MUTYH* variants. As such, it is difficult to make an association between breast cancer and MAP, and more studies are required to fully explore the association between the risk of breast cancer and the presence of biallelic *MUTYH* variants. Studies sequencing the entire *MUTYH* gene in breast cancer cohorts or investigating somatic G:C > T:A transversions in breast tumors of MAP patients could potentially fill the current knowledge gaps.

Endometrial cancers have also been reported in MAP patients, but like breast cancer, the association between endometrial cancer and MAP remains contested because there is no statistically significant increase of endometrial cancer in MAP patients [12, 20, 55, 57–59]. This association is based on three case reports. The first two describe a c.34G > T p.(Gly12Cys) transversion in the *KRAS* gene of MAP patients with endometrial cancer [59, 60]. One of these patients developed endometrial cancer at 46, despite the average age of diagnosis being 61 in the general population. While the early onset of endometrial cancer has been associated with Lynch syndrome [59], this patient's tumors were microsatellite stable and thus could not be the result of Lynch syndrome. The third case report described excess G:C > T:A transversions and the presence of the c.34G > T p.(Gly12Cys) variant in the *KRAS* gene in the endometrial cancer of a MAP patient [60]. These observations mean that endometrial cancer is likely to be associated with MAP, but investigations on a larger scale are required to fully validate this relationship [59].

Thyroid cancer has also been reported in MAP patients that have inherited at least one of the pathogenic founder variants [5, 12, 61–63]. Although the association between thyroid cancer and FAP has been reported [12], it is difficult to describe thyroid cancer as one of the manifestations of MAP, especially in the absence of or the lack of information about Gly12Cys variants in the *KRAS* gene.

Pulmonary bronchioloalveolar carcinoma (BAC) is another type of cancer that has been reported in connection with MAP [64], although only in a single patient. In this case, molecular analysis identified G:C > T:A transversions in *KRAS* in both CRC samples and seven of nine pulmonary BAC samples. The presence of these transversions suggests that it could be part of the MAP phenotypic spectrum, but like the previously described cancers, a larger dataset is needed to definitively confirm any link between BAC and MAP.

Desmoids have been reported in three MAP patients [22, 52, 53]. One of these patients had a mesenteric desmoid tumor, but the location of desmoids was not recorded for the others [53]. The patient with the mesenteric desmoid tumor was homozygous for p.(Gly396Asp) and had an aggressive phenotype, characterized by over 100 tubular adenomas in the colon and 4 CRCs by the age of 30. Osteomas have only been described in four MAP patients to date [4, 52, 65]. Because they are so rarely described in MAP, desmoids and osteomas might be distinguishing features between FAP and MAP [12]. Therefore, desmoid and osteomas are unlikely to be associated with MAP.

Very few studies have reported congenital hypertrophy of the retinal pigment epithelium (CHRPE) in MAP patients [4, 6, 12, 22, 38, 66]; however, it is unclear if these are the same type associated with FAP. In FAP patients, CHRPE is characterized by multiple bilateral, diffusely distributed, sharp bordered lesions. Sebaceous gland adenomas have also been reported multiple times in the literature [12, 39, 58, 61, 67, 68], which suggests that its association with CRC is not confined to patients with Lynch syndrome [12]. Other benign extraintestinal manifestations that have been reported in MAP patients thus far include dental cysts, dermoid cysts, jawbone cysts, hepatic cysts, kidney cysts, lipoma, benign endometrial tumors, and benign breast tumors [2, 4, 6, 12, 65].

## 4. Conclusion

While a phenotypic correlation with specific pathogenic variants was not observed, the founder variants carry an increased risk of CRC [6, 33, 34]. When compared to p.(Gly396Asp), the variant p.(Tyr179Cys) is associated with increased CRC and duodenal adenoma risk, earlier onset, and more severe phenotype. The term MAP implies that polyps are an obligatory phenotype, but numerous CRC cases in the absence of polyps suggest otherwise. There are also a multitude of extraintestinal manifestations associated with MAP or seen in MAP patients. Bladder, skin, ovarian and duodenal cancers, and adenomas have been found in multiple MAP patients, but the increased risk of developing endometrial, breast, and thyroid cancer in these patients remains controversial. Desmoids and osteomas are rarely described in MAP and thus might be features that distinguish MAP from FAP. There are several other benign extraintestinal manifestations reported in the literature, but their association with MAP has yet to be concretely established.

As discussed in our review, the phenotypic spectrum of MAP is much broader than polyposis alone and can manifest even as extracolonic cancers such as bladder and ovarian cancers. Hence, we would like to suggest the adoption of “*MUTYH*-associated tumor syndrome,” a term first described in Magrin et al. [11] to recognise the broader tumor phenotype. This new definition of MAP encompasses the extracolonic phenotypic spectrum caused by *MUTYH* deficiency while excluding the presence of polyps as a necessary criterion for diagnosis. Because there is an overlap between the clinical and molecular characteristics of *MUTYH*-associated tumor syndrome and Lynch syndrome, underlying constitutional variants in MMR genes must be ruled out before considering *MUTYH* deficiency as potential diagnosis. The somatic transversion that leads to the Gly12Cys variant in *KRAS* and *APC* is a well-documented consequence of *MUTYH* deficiency and could be used to firmly establish a causal relationship between MAP and the controversial phenotypes discussed here. This phenotypic review will facilitate the work of the InSiGHT-ClinGen Variant Curation Expert Panel as it outlines important phenotypic features of MAP for assessing variant pathogenicity and ultimately contribute to improved patient care.

## Figures and Tables

**Table 1:** Frequency of each variant type reported in Figure 2.

**Variant type**	**Number reported in literature, LOVD, and ClinVar**	**Number reported in literature with phenotype**
Missense	33	29
Nonsense	71	16
Frameshift	87	14
Splice site	27	10
In-frame insertion/deletion	7	6
Large deletion	9	2
Total	234	77

## Data Availability

Data is provided in the Supporting Information section.

## Nomenclature

ACMG: American College of Medical Genetics and Genomics
AMP: American Association of Molecular Pathology
APC: Adenomatous polyposis coli
BER: Base excision repair
CHRPE: Congenital hypertrophy of the retinal pigment epithelium
CRC: Colorectal cancer
FAP: Familial adenomatous polyposis
HP: Hyperplastic polyps
MAP: *MUTYH*-associated polyposis
MMR: Mismatch repair
SIR: Standardised incidence ratio
SSL: Sessile serrated lesions

## Supporting Information

Supporting Information Additional supporting information can be found online in the Supporting Information section. Table S1. Research strategy and results (6/6/2022–1540 AEST). Table S2. Clinical features of patients with biallelic MUTYH mutations.

## References

[1] Al-Tassan N., Chmiel N. H., Maynard J. (2002). Inherited variants of MYH associated with somatic G:C-->T:A mutations in colorectal tumors. *Nature Genetics*.

[2] Sampson J. R., Dolwani S., Jones S. (2003). Autosomal recessive colorectal adenomatous polyposis due to inherited mutations of MYH. *Lancet*.

[3] Nielsen M., Franken P. F., Reinards T. H. (2005). Multiplicity in polyp count and extracolonic manifestations in 40 Dutch patients with MYH associated polyposis coli (MAP). *Journal of Medical Genetics*.

[4] Gismondi V., Meta M., Bonelli L. (2004). Prevalence of the Y165C, G382D and 1395delGGA germline mutations of the MYH gene in Italian patients with adenomatous polyposis coli and colorectal adenomas. *International Journal of Cancer*.

[5] Aretz S., Uhlhaas S., Goergens H. (2006). MUTYH-associated polyposis: 70 of 71 patients with biallelic mutations present with an attenuated or atypical phenotype. *International Journal of Cancer*.

[6] Sieber O. M., Lipton L., Crabtree M. (2003). Multiple colorectal adenomas, classic adenomatous polyposis, and germ-line mutations in *MYH*. *New England Journal of Medicine*.

[7] Wang L., Baudhuin L. M., Boardman L. A. (2004). MYH mutations in patients with attenuated and classic polyposis and with young-onset colorectal cancer without polyps. *Gastroenterology*.

[8] Avezzù A., Agostini M., Pucciarelli S. (2008). The role of MYH gene in genetic predisposition to colorectal cancer: another piece of the puzzle. *Cancer Letters*.

[9] Balaguer F., Castellví-Bel S., Castells A. (2007). Identification of MYH mutation carriers in colorectal cancer: a multicenter, case-control, population-based study. *Clinical Gastroenterology and Hepatology*.

[10] Croitoru M. E., Cleary S. P., Di Nicola N. (2004). Association between biallelic and monoallelic germline MYH gene mutations and colorectal cancer risk. *Journal of the National Cancer Institute*.

[11] Magrin L., Fanale D., Brando C. (2022). MUTYH-associated tumor syndrome: the other face of MAP. *Oncogene*.

[12] Vogt S., Jones N., Christian D. (2009). Expanded extracolonic tumor spectrum in MUTYH-associated polyposis. *Gastroenterology*.

[13] Jones S., Emmerson P., Maynard J. (2002). Biallelic germline mutations in *MYH* predispose to multiple colorectal adenoma and somatic G:C→T:A mutations. *Human Molecular Genetics*.

[14] Venesio T., Molatore S., Cattaneo F., Arrigoni A., Risio M., Ranzani G. N. (2004). High frequency of MYH gene mutations in a subset of patients with familial adenomatous polyposis. *Gastroenterology*.

[15] Isidro G., Laranjeira F., Pires A. (2004). Germline MUTYH (MYH) mutations in Portuguese individuals with multiple colorectal adenomas. *Human Mutation*.

[16] Abdelmaksoud-Dammak R., Miladi-Abdennadher I., Amouri A. (2012). High prevalence of the c.1227_1228dup (p.Glu410GlyfsX43) mutation in Tunisian families affected with MUTYH-associated-polyposis. *Familial Cancer*.

[17] Lefevre J. H., Colas C., Coulet F. (2011). Frequent mutation in North African patients with MUTYH-associated polyposis. *Clinical Genetics*.

[18] Kdissa A., Brusgaard K., Ksiaa M. (2020). c.1227_1228dupGG (p.Glu410Glyfs), a frequent variant in Tunisian patients with MUTYH associated polyposis. *Cancer Genetics*.

[19] Nielsen M., Joerink - van de Beld M. C., Jones N. (2009). Analysis of MUTYH genotypes and colorectal phenotypes in patients with MUTYH-associated polyposis. *Gastroenterology*.

[20] Guarinos C., Juárez M., Egoavil C. (2014). Prevalence and characteristics of MUTYH-associated polyposis in patients with multiple adenomatous and serrated polyps. *Clinical Cancer Research*.

[21] Theodoratou E., Campbell H., Tenesa A. (2010). A large-scale meta-analysis to refine colorectal cancer risk estimates associated with MUTYH variants. *British Journal of Cancer*.

[22] Filipe B., Baltazar C., Albuquerque C. (2009). APC or MUTYH mutations account for the majority of clinically well-characterized families with FAP and AFAP phenotype and patients with more than 30 adenomas. *Clinical Genetics*.

[23] Fostira F., Papademitriou C., Efremidis A., Yannoukakos D. (2010). An in-frame exon-skipping MUTYH mutation is associated with early-onset colorectal cancer. *Diseases of the Colon & Rectum*.

[24] Aceto G., Curia M. C., Veschi S. (2005). Mutations of *APC* and *MYH* in unrelated Italian patients with adenomatous polyposis coli. *Human Mutation*.

[25] Lipton L., Halford S. E., Johnson V. (2003). Carcinogenesis in MYH-associated polyposis follows a distinct genetic pathway. *Cancer Research*.

[26] Jones S., Lambert S., Williams G. T., Best J. M., Sampson J. R., Cheadle J. P. (2004). Increased frequency of the k-ras G12C mutation in MYH polyposis colorectal adenomas. *British Journal of Cancer*.

[27] Thomas L. E., Hurley J. J., Meuser E. (2017). Burden and profile of somatic mutation in duodenal adenomas from patients with familial adenomatous-and MUTYH-associated polyposis. *Clinical Cancer Research*.

[28] Nielsen M., de Miranda N. F., van Puijenbroek M. (2009). Colorectal carcinomas in MUTYH-associated polyposis display histopathological similarities to microsatellite unstable carcinomas. *BMC Cancer*.

[29] Huang L., Guo Z., Wang F., Fu L. (2021). KRAS mutation: from undruggable to druggable in cancer. *Signal Transduction and Targeted Therapy*.

[30] Viel A., Bruselles A., Meccia E. (2017). A specific mutational signature associated with DNA 8-oxoguanine persistence in MUTYH-defective colorectal cancer. *EBioMedicine*.

[31] van Puijenbroek M., Nielsen M., Tops C. M. (2008). Identification of patients with (atypical) MUTYH-associated polyposis by KRAS2 c.34G > T prescreening followed by MUTYH hotspot analysis in formalin-fixed paraffin-embedded tissue. *Clinical Cancer Research*.

[32] Georgeson P., Harrison T. A., Pope B. J. (2022). Identifying colorectal cancer caused by biallelic MUTYH pathogenic variants using tumor mutational signatures. *Nature Communications*.

[33] Jenkins M. A., Croitoru M. E., Monga N. (2006). Risk of colorectal cancer in monoallelic and biallelic carriers of MYH mutations: a population-based case-family study. *Cancer Epidemiology, Biomarkers & Prevention*.

[34] Farrington S. M., Tenesa A., Barnetson R. (2005). Germline susceptibility to colorectal cancer due to base-excision repair gene defects. *American Journal of Human Genetics*.

[35] Kanter-Smoler G., Björk J., Fritzell K. (2006). Novel findings in Swedish patients with MYH-associated polyposis: mutation detection and clinical characterization. *Clinical Gastroenterology & Hepatology*.

[36] Olschwang S., Blanché H., de Moncuit C., Thomas G. (2007). Similar colorectal cancer risk in patients with monoallelic and biallelic mutations in the MYH gene identified in a population with adenomatous polyposis. *Genetic Testing*.

[37] Lefevre J. H., Parc Y., Svrcek M. (2009). APC, MYH, and the correlation genotype-phenotype in colorectal polyposis. *Annals of Surgical Oncology*.

[38] Jo W. S., Bandipalliam P., Shannon K. M. (2005). Correlation of polyp number and family history of colon cancer with germline MYH mutations. *Clinical Gastroenterology & Hepatology*.

[39] Morak M., Laner A., Bacher U., Keiling C., Holinski-Feder E. (2010). MUTYH-associated polyposis - variability of the clinical phenotype in patients with biallelic and monoallelic MUTYH mutations and report on novel mutations. *Clinical Genetics*.

[40] Dallosso A. R., Dolwani S., Jones N. (2008). Inherited predisposition to colorectal adenomas caused by multiple rare alleles of MUTYH but not OGG1, NUDT1, NTH1 or NEIL 1, 2 or 3. *Gut*.

[41] Patel R., McGinty P., Cuthill V. (2020). MUTYH-associated polyposis - colorectal phenotype and management. *Colorectal Disease*.

[42] Grover S., Kastrinos F., Steyerberg E. W. (2012). Prevalence and phenotypes of APC and MUTYH mutations in patients with multiple colorectal adenomas. *Journal of the American Medical Association*.

[43] Russell A. M., Zhang J., Luz J. (2006). Prevalence of MYH germline mutations in Swiss APC mutation‐negative polyposis patients. *International Journal of Cancer*.

[44] Boparai K. S., Dekker E., Van Eeden S. (2008). Hyperplastic polyps and sessile serrated adenomas as a phenotypic expression of MYH-associated polyposis. *Gastroenterology*.

[45] Nielsen M., Morreau H., Vasen H. F., Hes F. J. (2011). MUTYH-associated polyposis (MAP). *Critical Reviews in Oncology/Hematology*.

[46] Zorcolo L., Fantola G., Balestrino L. (2011). MUTYH-associated colon disease: adenomatous polyposis is only one of the possible phenotypes. A family report and literature review. *Tumori*.

[47] Buchanan D., Young J. (2009). A perspective on bi-allelic MUTYH mutations in patients with hyperplastic polyposis syndrome. *Gastroenterology*.

[48] Nielsen M., Poley J. W., Verhoef S. (2006). Duodenal carcinoma in MUTYH-associated polyposis. *Journal of Clinical Pathology*.

[49] Walton S. J., Kallenberg F. G., Clark S. K., Dekker E., Latchford A. (2016). Frequency and features of duodenal adenomas in patients with MUTYH-associated polyposis. *Clinical Gastroenterology & Hepatology*.

[50] Collaborative Group on Duodenal Polyposis in MAP, Thomas L. E., Hurley J. J. (2021). Duodenal adenomas and cancer in MUTYH-associated polyposis: an international cohort study. *Gastroenterology*.

[51] D'Elia G., Caliendo G., Casamassimi A., Cioffi M., Molinari A. M., Vietri M. T. (2018). APC and MUTYH analysis in FAP patients: a novel mutation in APC gene and genotype-phenotype correlation. *Genes*.

[52] Yanaru-Fujisawa R., Matsumoto T., Ushijima Y. (2008). Genomic and functional analyses of MUTYH in Japanese patients with adenomatous polyposis. *Clinical Genetics*.

[53] de Ferro S. M., Suspiro A., Fidalgo P. (2009). Aggressive phenotype of MYH-associated polyposis with jejunal cancer and intra-abdominal desmoid tumor: report of a case. *Diseases of the Colon & Rectum*.

[54] Liu C., Matsukuma K., Tejaswi S. (2022). Gastric pyloric gland adenoma in MUTYH-associated polyposis. *Clinical Gastroenterology & Hepatology*.

[55] Win A. K., Reece J. C., Dowty J. G. (2016). Risk of extracolonic cancers for people with biallelic and monoallelic mutations in MUTYH. *International Journal of Cancer*.

[56] Beiner M. E., Zhang W. W., Zhang S., Gallinger S., Sun P., Narod S. A. (2009). Mutations of the MYH gene do not substantially contribute to the risk of breast cancer. *Breast Cancer Research and Treatment*.

[57] Gilpin C., Lines M., Tomiak E. (2011). When is a desmoid not a desmoid? Endometrial cancer as an extracolonic manifestation of MYH associated polyposis (MAP). *Hereditary Cancer in Clinical Practice*.

[58] Barnetson R. A., Devlin L., Miller J. (2007). Germline mutation prevalence in the base excision repair gene, MYH, in patients with endometrial cancer. *Clinical Genetics*.

[59] Tricarico R., Bet P., Ciambotti B. (2009). Endometrial cancer and somatic G>T KRAS transversion in patients with constitutional MUTYH biallelic mutations. *Cancer Letters*.

[60] Villy M. C., Masliah-Planchon J., Buecher B. (2022). Endometrial cancer may be part of the MUTYH-associated polyposis cancer spectrum. *European Journal of Medical Genetics*.

[61] Ponti G., Ponz de Leon M., Maffei S. (2005). Attenuated familial adenomatous polyposis and Muir-Torre syndrome linked to compound biallelic constitutional MYH gene mutations. *Clinical Genetics*.

[62] Pervaiz M. A., Eppolito A., Schmidt K. (2010). Papillary thyroid cancer in a patient with MUTYH-associated polyposis (MAP). *Familial Cancer*.

[63] Wang M., Zhu F., Luo N., Han T., Wang M. (2021). A case report of a patient with first phenotype of papillary thyroid carcinoma and heterochronous multiprimary tumor harboring germline MUTYH Arg19/Gly286Glu mutations. *Oral Oncology*.

[64] von der Thüsen J. H., van de Wetering M. D., Westermann A. M., Heideman D. A., Thunnissen E. (2011). Bronchioloalveolar adenocarcinoma and pulmonary Langerhans cell histiocytosis in a patient with MUTYH-associated polyposis. *Journal of Clinical Oncology*.

[65] Kairupan C. F., Meldrum C. J., Crooks R. (2005). Mutation analysis of the MYH gene in an Australian series of colorectal polyposis patients with or without germline APC mutations. *International Journal of Cancer*.

[66] Sutcliffe E. G., Bartenbaker Thompson A., Stettner A. R. (2019). Multi-gene panel testing confirms phenotypic variability in MUTYH-associated polyposis. *Familial Cancer*.

[67] Ajith Kumar A., Gold J. A., Mallon E., Thomas S., Hodgson S. V. (2008). Sebaceous adenomas in an MYH associated polyposis patient of Indian (Gujarati) origin. *Familial Cancer*.

[68] Kacerovska D., Drlik L., Slezakova L. (2016). Cutaneous sebaceous lesions in a patient with MUTYH-associated polyposis mimicking Muir-Torre syndrome. *American Journal of Dermatopathology*.

[69] Thet M., Plazzer J. P., Capella G. (2024). Phenotype correlations with pathogenic DNA variants in the *MUTYH* gene. *medRxiv*.

