# Supplementary material for: Phenotype Correlations With Pathogenic DNA Variants in the MUTYH Gene: A Review of Over 2000 Cases
Source: Hum Mutat. 2024 Sep 27;2024:8520275. doi: 10.1155/2024/8520275 (PMC11918913; doi:10.1155/2024/8520275)
Supplement: Supporting Information — Additional supporting information can be found online in the Supporting Information section. Table S1. Research strategy and results (6/6/2022–1540 AEST). Table S2. Clinical features of patients with biallelic MUTYH mutations. [file 8520275.f1.docx]

# Supplemental Section

**Table S1. Research Strategy and Results (6/6/2022 – 15:40 AEST).**

| Search terms | | Number of results | |
| --- | --- | --- | --- |
|  |  | MEDLINE | EMBASE |
|  | MUTYH or MUTYH gene or MYH or hMYH or MutY homolog or MUTYH protein or mutY adenine glycosylase or MUTYH deficiency or MUTYH mutation or MUTYH-associated polyposis or base excision repair | 6654 | 9341 |
|  | Adenomatous polyp* or polyp* or MUTYH-associated polyp* or MUTYH-associated tumour* or colorectal cancer* or colorectal carcinoma* or colorectal tumour* or colorectal adenoma* or gastrointestinal cancer* or gastrointestinal carcinoma* or gastrointestinal tumour* or extracolonic cancer* or extracolonic carcinoma* or extracolonic tumour* or extraintestinal cancer* or extraintestinal carcinoma* or extraintestinal tumour* | 481353 | 743957 |
|  | Phenotypic variabilit* or phenotype or genotype-phenotype correlation* or genetic association stud* | 567693 | 848086 |
|  | 2 or 3 | 1033312 | 1564902 |
|  | 1 and 4 | 1285 | 2259 |
|  | (6).ti | 277 | 382 |
|  | Limit 6 to (human and English language) | 245 | 327 |

**Table S2. Clinical Features of Patients with Biallelic *MUTYH* Mutations.**

| **Article** | **Geographical area**  **(Biallelic *MUTYH/* study population*)** | **Mean/median age of onset (range) in years** | **Polyp count** | **CRC** | **Mean age of CRC onset (range) in years** | **Duodenal lesions^a^** | **Gastric lesions** | **Malignant extraintestinal manifestations** | **Benign extraintestinal manifestations** |
| --- | --- | --- | --- | --- | --- | --- | --- | --- | --- |
| Sampson 2003 (2) | UK   25 /111 (22.5%) | 46/ 48 (13-65) | >100 = 9  10-100 = 11 multiple/ numerous = 5 | 12/25 (48%) | 49.7 | Duodenal adenomas 1/25 (4%) | Gastric cancer (17y M) | Investigations/ findings not reported | Investigations/ findings not reported |
| Nielsen 2005 (3) | The Netherlands  40/170 (23.5%) | 45/ 45 (21-67) | 10-99 = 24/83 (29%) 100-1000 = 7/24 (29%) | 26/40 (65%) 16/26 Right sided | 47 | Duodenal cancer 1/16 (6.25%)  Duodenal adenomas 5/16 (31%)  Barrett oesophagus with carcinoma 1/16 (6.25%) | Fundic gland polyps 1/16 (6.25%) | Breast cancer 4/22 (18%)  Cervical cancer 1/22 (4.5%)  Basal cell carcinoma 2/40 (5%)  Bladder cancer 1/40 (2.5%) | Investigations/ findings not reported |
| Gismondi 2004 (4) | Italy  FAP: 5/38 cases with ≥100 polyps (13.2%)  AAPC: 8/31 cases with 10-99 polyps (25.8%)  0/141 cases with <10 polyps  0/52 controls without any polyps | 10-99 polyps group: 47/ 48.5 (27-59)  ≥100 polyps group: 48.6/ 49 (37-58) | 10-99 = 8 ≥100 = 5 | 10-99 = 6/8 (75%)  ≥100 = 4/5 (80%) | - | Investigations/ findings not reported | Investigations/ findings not reported | Not reported | 10-99 polyp group:  CHRPE 1/8 (12.5%)  Osteomas 2/8 (25%)  ≥100 polyp group:  CHRPE 2/5 (40%)  Dermoid cysts 1/5 (20%)  Dental cysts 1/5 (20%) |
| Aretz 2006 (5) | Germany  55/329 cases (16.7%) + 9 index cases + 7 affected relatives | 45 (24-72) | 15-100 = 17  >100 = 8  Multiple = 13  Unknown = 17 | 28/56 (50%) | 48 (29-72) | Duodenal polyps 6/33 (18%) | Stomach cancer 1/33 (3.03%) | Thyroid cancer 1/71 (1.4%) | Lipoma 2/71 (2.8%)^b^ |
| Sieber 2003 (6) | UK  3-100 polyps: 6/152 (4%) >100 polyps: 8/107 (7.5%) | <100: 56 (45-59)  100-1000: 48 (30-70) | <100 = 6  100-1000 = 8 | <100: 3/6 (50%)  100-1000: 4/8 (50%) | - | Duodenal adenomas 2/8 (25%)^c^ | Not reported | Investigations/ findings not reported | CHRPE 1/8 (12.5%) ^c^ |
| Wang 2004 (7) | USA  16/140 patients with 4-500 polyps (11.4%)  2/116 patients with CRC ≤50yo (1.7%) | 46.9 (37-62) | No polyp = 1  <20 = 1  20-99 = 6  100-499 = 4  Multiple = 4  Clinical FAP^d^ = 1 Unknown = 1 | 2/16 (12.5%) | - | Investigations/ findings not reported | Investigations/ findings not reported | Investigations/ findings not reported | Investigations/ findings not reported |
| Avezzu 2008 (8) | Italy  2/439 (0.46%) | Biallelic MUTYH mutations were found in 2/439 patients. Both had colorectal cancer. One had diffuse polyps and the other only had one polyp. No other phenotype was investigated or reported. | | | | | | | |
| Balaguer 2007 (9) | Spain  8/1116 (0.72%) | 58/ 56.5 (45-73) | No polyp = 2  1 polyp = 2  20-100 = 4 | n/a^e^ | n/a | Investigations/ findings not reported | Investigations/ findings not reported | Breast cancer 1/8 (12.5%)  Sarcoma 1/8 (12.5%) | Investigations/ findings not reported |
| Croitoru 2004 (10) | Canada  12/1238 (0.97%) | 12/1238 case patients had biallelic MUTYH mutations, of which all of them had CRC and 3 did not have polyps. The study also found that the mean age of diagnosis of CRC in patients with p.(Tyr179Cys) and/or p.(Gly396Asp) is younger than those without these mutations. Moreover, patients with p.(Tyr179Cys) and/or p.(Gly396Asp) are associated with double the risk of CRC. No other phenotype was investigated or reported. | | | | | | | |
| Vogt 2009 (12) | UK, Germany, the Netherlands  276 MAP patients (158M, 118F) | 45 (12-70) | - | 152/276 (55%) | 48.6/48 (21-70) | Duodenal polyps 26/150 (17%)  Duodenal cancer 2/150 (1.3%) | Gastric cancer 3/150 (2%) Gastric polyps 17/150 (11%) Oesophageal carcinoma 2/150 (1.5%) | Bladder cancer 4/276 (1.5%)  Skin cancer^f^ 13/276 (4.7%)  Lung cancer 2/276 (0.7%)  Breast cancer ^g^ 8/276 (2.9%)  Ovarian cancer 3/118 (2.5%)  Endometrial cancer 2/118 (1.7%) | Sebaceous gland adenoma 5/276 (1.8%)  Epidermoid cysts 3/276 (1%)  Lipoma 8/276 (3%)  Jaw-bone cysts 11/276 (4%)  Hepatic cysts 5/276 (1.8%)  Kidney cysts 2/276 (0.7%)  Benign endometrial tumour 4/118 (3.4%)  Benign breast tumours 4/118 (3.4%) |
| Venesio 2004 (14) | Italy  5/14 | 53.8/ 58 (35-65) | 30-100 = 5 | 2/5 (40%) | - | Investigations/ findings not reported | Investigations/ findings not reported | Investigations/ findings not reported | Investigations/ findings not reported |
| Isidro 2004 (15) | Portugal  21/53 (39.6%) | 50.4/ 50 (36-68) | <20 = 1  20-99 = 14 >100 = 6 | 13/21 (61.9%) | - | Not reported | Not reported | Breast cancer 1/53 (1.9%) | Not reported |
| Lefevre 2011 (17) | North Africa  42 MAP patients | c.1227_1228dup: 44.3 (30-62)  Other mutations: 44.2 (24-60) | >30 = 34/42 | 31/42 (73.8%) | - | Duodenal polyps 7/26 (26.9%) | Investigations/ findings not reported | Investigations/ findings not reported | Investigations/ findings not reported |
| Nielsen 2009 (19) | The Netherlands 257 MAP patients | 45 (12-68) | - | 148/254 (58%)  77/138 (56%) right sided | 48 (21-70) | Investigations/ findings not reported | Investigations/ findings not reported | Investigations/ findings not reported | Investigations/ findings not reported |
| Guarinos 2014 (20) | Spain  27/405 patients (6.7%) | 52.7/ 53 (34-75) | <20 = 4  20-99 = 19  >100 = 4 | 16/27 (59.3%)  9/15 known location right sided | - | Investigations/ findings not reported | Investigations/ findings not reported | Endometrial cancer^h^ (2)  Ovarian cancer (1)  Testicular cancer (1) | Investigations/ findings not reported |
| Filipe 2009 (22) | Portugal  21/107 patients (19.6%) | 46 (23-69) | 10-29 = 2  30-99 = 7 | 15/21 (71%) | - | Duodenal tumour 2/21 (9.52%) | Fundic gland polyps 1/21 (4.76%) | Not reported | Desmoid 1/21 (4.76%)  Thyroid tumours 1/21 (4.76%)  Sebaceous cyst 1/21 (4.76%)  CHRPE 1/21 (4.76%) |
| Dallosso 2008 (40) | UK and New Zealand  33/167 (19.8%) | 47/ 48 (13-67) | <10 = 1  10-100 = 15  >100 = 10 Multiple/ numerous = 7 | 19/33 (58%) | 50 | Observed (not quantified; inconsistent screening for upper GI disease in this study) | Gastric adenomas 1/9 (11.11%) | Breast cancer 1/9 (11.11%) | Investigations/ findings not reported |
| Lipton 2003 (25) | UK, Finland, Denmark, Switzerland  22 MAP patients | 55/ 55 (38-71) | <20 = 3  20-100 = 7  >100 = 3  Multiple/ numerous/ several = 6  Not reported = 3 | 18/ 22 (81.8%)  11/16 known location left sided | - | Not reported | Not reported | Not reported | Not reported |
| Patel 2020 (41) | UK  134 MAP patients | - | - | 68/134 (50.7%)  43/81 (53%) of tumours right sided | 47 (33-74) | Not investigated | Not investigated | Not investigated | Not investigated |
| Russell 2006 (43) | Switzerland  7/79 APC-negative patients (8.9%) | 45.6/ 48 (33-60) | <100 = 6  >100 = 1 | 5/7 (71.4%) | - | Duodenal adenomas 1/7 (14.3%) | Not reported | Not reported | Not reported |
| Jo 2005 (38) | USA  7/45 patients with ≥15 polyps (15.6%) + 2 MAP patients with <15 polyps | 44.4/43 (33-53) | <15 = 2 20-100 = 6 >100 = 1 | 5/9 (55.6%) | 41.3 (33-46)^h^ | Duodenal adenomas 2/9 (22.2%) | Fundic gland polyps 3/9 (33.3%) | Breast cancer^i^ (1) | CHRPE 1/9 (11.1%) |
| Morak 2010 (39) | Germany  33/215 patients (15.3%) | 43 (28-64) | <10 = 6  10-100 = 16  >100 = 11 | 11/33 (33%) | 43 (29-64) | Duodenal polyps + gastric polyps 10/33 (30%) Gall bladder polyp (1/33) | Duodenal polyps + gastric polyps 10/33 (30%) | Not reported | Not reported |
| Kanter-Smoler 2006 (35) | Switzerland  6/15 patients | 48/51 (23-59) | 20-100 = 2 100-1000 = 2 >1000 = 1 Multple = 1 | 4/6 (66.7%) all right sided | - | Duodenal adenomas 1/6 (16.7%) | Not reported | Investigations/ findings not reported | Investigations/ findings not reported |
| Olschwang 2007 (36) | France  49/406 patients (12.1%) | 47 | 5-14 = 18  15-99 = 18  ≥100 = 13 | 42/49 (85.7%) 60% in the proximal colon | 51 | Duodenal adenocarcinoma 2/49 (4.1%) | Gastric cancer 1/49 (2%)  Fundic gland polyps 1/49 (2%) | Breast cancer 1/49 (2%)  Uterine cancer 1/49 (2%) Basocellular carcinoma 1/49 (2%)  Central nervous system 1/49 (2%) | Not reported |
| Walton 2016 (49) | the Netherlands, UK  92 MAP patients | - | - | - | - | Duodenal adenomas 31/92 (34%) | Not reported | Investigations/ findings not reported | Investigations/ findings not reported |
| Thomas 2021(50) | UK  394 MAP patients | - | - | 211/394 (54.4%) | - | Duodenal adenomas 57/394 (21.1%) | Stomach cancer 2/394 (0.5%)  Fundic gland polyps (not quantified) | Endometrial cancer ^j^ (9) | Investigations/ findings not reported |
| Daans 2020 (69) | The Netherlands  94 MAP patients | - | - | - | - | Barrett’s oesophagus 7/72 (9.7%)  Oesophageal adenocarcinoma 1/72 (1.4%) | Not reported | Investigations/ findings not reported | Investigations/ findings not reported |
| Enholm 2003 (70) | Finland  4/1042 CRC patients (0.4%) | 53.25/ 53.5 (40-66) | 50-100 = 2 5 adenomas = 1  Several = 1 | All patients in the study population had CRC | - | Investigations/ findings not reported | Investigations/ findings not reported | Melanoma 1/4 (25%) | Not reported |
| Farrington 2005 (34) | Scotland  12/2239 cases (0.5%) | - | 0 polyps = 4/11 (36%)  >0 = 7/11 (64%) | 12/12 (100%) | - | Investigations/ findings not reported | Investigations/ findings not reported | Investigations/ findings not reported | Investigations/ findings not reported |
| Grover 2012 (42) | USA  422/8676 patients (4.9%) | 47 (39-52) | Missing = 4/401 (4%)  <10 = 19/401 (5%)  10-19 = 37/401 (9%)  20-99 = 233/401 (58%)  100-999 = 94/401 (23%)  ≥1000 = 2/401 (0.5%) | 162/422 (38.3%) | 46 (39-52) | Investigations/ findings not reported | Investigations/ findings not reported | -^k^ (See footnote) | -^k^ (See footnote) |
| Kairupan 2005 (65) | Australia  5/32 patients (15.6%) + 36 relatives who have biallelic *MUTYH* mutations | 45.5 | <5 = 2  5-19 = 6  20-100 = 14  >100 = 6  Unknown = 13 | 18/41 (43.9%) | - | Duodenal polyp 1/41 (2.4%) | Not reported | Melanoma 1/41 (2.4%) | Jaw exostosis (osteoma) 1/47 (2.1%)  Sebaceous cysts 1/47 (2.1%) |
| Kim 2007 (71) | Korean  2/62 patients (3.2%) | 48.5 | 14-99 = 2 | 2/2 (100%) | - | Not reported | Not reported | Not reported | Not reported |
| Lefevre 2009 (37) | France  17/384 patients (4.4%) | - | >100 = 6 | 13/17 (76.5%) | - | Duodenal polyps 3/17 (21.4%) | Gastric glandular polyps 2/17 (14.3%) | Not reported | Desmoid 0/17 |
| Miyaki 2005 (72) | Japanese  1/35 patients (2.9%) | 41 | >100 = 1 | 1/1 (100%) | 41 | Not reported | Not reported | Not reported | Not reported |
| Sutcliffe 2019 (66) | USA  82 MAP patients | 49.2 | 20-99 = 33/61  >100 = 9/61  10-19 = 13/61  <10 = 6/61 | 40/82 (488%) | 49.4 | Not reported | Fundic gland polyps 1/82 (1.2%) | Breast cancer 7/45 15.6%)  Endometrial cancer 4/45 (8.9%)  Non-melanoma skin cancer (5/82 (6.1%)  Melanoma 2/82 (2.4%)  Sebaceous neoplasm 2/82 (2.4%)  Pancreatic cancer 2/82 (2.4%)  Head and neck cancer 2/82 (2.4%)  Others^l^ 6/82 (7.3%) | CHRPE 2/82 (2.4%) |
| Yanaru-Fujisawa 2008 (52) | Japanese  9/66 patients with >100 polyps (13.6%) | 45.4/43 (24-66) | >100 = 7  >1000 = 2 | 5/9 (55.6%) | - | Duodenal adenomas 3/9 (33.3%) | Gastric cancer 2/9 (22.2%)  Gastric adenoma 2/9 (22.2%)  Fundic gland polyps 1/9 (11.1%) | Not reported | Lipoma 1/9 (11.1%)  Osteoma 1/9 (11.1%)  Desmoid 1/9 (11.1%) |
| **Summary statistics** | - | - | - | 1123/2109 (53.2%) | - | Duodenal adenomas 160/936 (17.1%)  Duodenal cancer 3/936 (0.3%) | - | - | - |

*Recruitment criteria varies across the studies, with some studies recruiting patients with CRC without *APC/MMR* genes and others only including patients with biallelic *MUTYH* mutations. ^a^Percentage calculated out of proportion of patients who underwent upper gastrointestinal endoscopy. ^b^Most patients have not been systematically examined for benign lesions. ^c^Extracolonic manifestations only reported for the patient group with >100 adenomas. ^d^Polyp count not defined. ^e^The entire study population had CRC, of which 8/1116 (0.7%) had biallelic constitutional mutations. ^f^inlcude basal cell carcinoma, melanoma and spinous cell carcinoma. ^g^Breast cancer found in 7/118 female patients and 1/158 male patients. ^h^mean calculated from data for 6/9 MAP patients. ^i^Number of male vs female MAP patients not reported. ^j^Other cancers in 49/394 (12.4%) patients including 9 endometrial cancer and 2 stomach cancer. Number of female cases not specified. ^k^517/8676 patients have extraintestinal manifestations but the details were not reported. ^l^Others include bladder, cervical, lung, ovarian, prostate and testicular.

In this table, “not reported” was used if a study investigated for a particular phenotype that was not found in the patients, “not investigated” was used if it was explicitly stated that the phenotype was not investigated, and “investigations/findings not reported” if there was no mention of investigation of the phenotype in the study.
